# Spinocerebellar Ataxia 36: From Mutations Toward Therapies

**DOI:** 10.3389/fgene.2022.837690

**Published:** 2022-03-04

**Authors:** Samuel Lopez, Fang He

**Affiliations:** Department of Biological and Health Sciences, Texas A&M University, Kingsville, TX, United States

**Keywords:** SCA36, GGCCTG, repeat expansion disorders, RAN translation, antisense oligos, motor neuron disease

## Abstract

Spinocerebellar ataxia 36 (SCA36) is a type of repeat expansion-related neurodegenerative disorder identified a decade ago. Like other SCAs, the symptoms of SCA36 include the loss of coordination like gait ataxia and eye movement problems, but motor neuron-related symptoms like muscular atrophy are also present in those patients. The disease is caused by a GGCCTG hexanucleotide repeat expansion in the gene *Nop56*, and the demographic incidence map showed that this disease was more common among the ethnic groups of Japanese and Spanish descendants. Although the exact mechanisms are still under investigation, the present evidence supports that the expanded repeats may undergo repeat expansion-related non-AUG-initiated translation, and these dipeptide repeat products could be one of the important ways to lead to pathogenesis. Such studies may help develop potential treatments for this disease.

## Introduction

Spinocerebellar ataxias (SCAs) are a group of autosomal dominant neurodegenerative diseases that share the key features of progressive cerebellar ataxia. Many different causative genetic mutations were identified in these diseases, and such mutations can be largely classified into two subgroups: those with nucleotide repeat expansions and those with point mutations (Ashizawa et al., 2018). SCA36 is a member of SCAs caused by nucleotide repeat expansions. Specifically, it is caused by a GGCCTG repeat expansion located at the host gene *Nop56* (Kobayashi et al., 2011)*.* This disease was first described in 2007 in families of a restricted region in Japan (Ohta et al., 2007), and further studies revealed that the incidence of this disease was among different ethnic groups from both the eastern and the western countries (García-Murias et al., 2012; Obayashi et al., 2015). In this minireview, we attempted to summarize the recent findings about the geographic locations, symptoms, genetic aspects, and pathogenesis and therapeutic interventions of this disease, with a hope to shed light and provide directions for future studies.

## Geographic Presence

### Incidence in Japan and Other Asian Areas

The initial identification of SCA36 came from the case studies of two patients in two unrelated pedigrees in Japan. The patients neither showed point mutations nor were there any nucleotide repeat expansions detected in prior known genes related to SCAs (Ohta et al., 2007). Later, repeat-primed PCR was developed to detect the GGCCTG repeat presence in the first intron of the *Nop56* gene, and nine families of a cohort of 251 SCA patients were found to carry the repeat expansion (Kobayashi et al., 2011). Furthermore, the analyses of more SCA patients revealed additional families that all originated from the Asidan river area in Japan, and thus, it was given the nickname Asidan by those researchers (Abe and Ikeda, 2012).

Several other Asian research groups also screened for the presence of GGCCTG repeat expansion in SCA patients. From the mainland of China, one study identified a total 1.6% of autosomal dominant SCA pedigrees (8/364) and 0.32% (1/312) patients of sporadic ataxias to carry the expanded GGCCTG repeats (Zeng et al., 2016), while another study reported that out of 204 pedigrees, no SCA36 cases were found (Peng et al., 2020). In the Taiwan area, Lee *et al.* found that the SCA36 incidence was 0.6% (3/512 pedigrees) in Han Chinese with ataxias (Lee et al., 2016).

### Incidence in Spain and Other European Areas

In a 2012 study in Spain, Garcia-Murias *et al.* found that the SCA36 repeat expansion is relatively common in a small Atlantic coastal area called Galicia in northwestern Spain, which gave it the name “Costa da Morte,” a Spanish expression meaning “coast of death” (García-Murias et al., 2012). Apart from the initial two large families, the screening of another 158 ataxia kindreds identified an additional eight families with the expanded SCA36 repeats, giving the total possible incidence of SCA36 to be around 6.3% out of all ataxias in this Galicia region.

In a multicenter research study from France, Japan, and Germany in 2015, Obayashi *et al.* found that 12 families from France (1.9% of all French SCAs), including one family each with Spanish, Portuguese, or Chinese ancestry, and five families from Japan (1.5% of all Japanese SCAs) had the expanded GGCCTG repeats of SCA36, while none of the German patients carried such repeat expansions (Obayashi et al., 2015). Another report in Poland found that five families had GGCCTG repeats from their study cohort (Sulek et al., 2013). Apart from these positive reports of SCA36 incidents, there have also been some negative reports from Europe in Germany, the United Kingdom, Greece, and Portugal (Hersheson et al., 2012; Loureiro et al., 2013; Sulek et al., 2013; Aydin et al., 2018; Katsimpouris et al., 2019).

### Incidence in the United States

One report in 2017 found that four out of 577 (0.7%) of undiagnosed ataxia is caused by GGCCTG repeats in the *Nop56* gene, and the origins were shown to be from European, Japanese, and Vietnamese ancestors (Valera et al., 2017).

The available analyses of ataxia patients from the globe clearly showed that the two concentrated regions of SCA36s would be located in eastern Asian (China and Japan mostly) and European (Spain and their neighboring countries, including France) areas. The disease is likely seen in immigrants overseas from these regions, while other regions of the world were either sparse or void of the presence. It would be very interesting to see whether these two areas share any common regions of SNP markers, so it can discern whether they were derived from a shared single founder or they were mutated independently.

## Clinical Symptoms

The initial symptoms of SCA36 patients usually show up at late 40s to early 50s (Kobayashi et al., 2011; García-Murias et al., 2012), but the age of onset (AOO) can be as early as late 30s (Obayashi et al., 2015). Often the first signs are gait ataxia, dizziness, or hearing loss (Kobayashi et al., 2011; García-Murias et al., 2012; Obayashi et al., 2015). The diseases showed an apparent autosomal dominant inheritance mode such as other SCAs, with no clear sex differences (Kobayashi et al., 2011; García-Murias et al., 2012; Obayashi et al., 2015). Some typical symptoms are summarized later.

### Common Movement Coordination Signs

The early symptoms from SCA36 patients were often very similar to other SCA patients, such as unsteady gait and action tremor (Ohta et al., 2007). Patients with SCA36 showed slow progression of disease and eventually developed the difficulty in speaking (dysarthria). Another common feature in SCA36 patients was abnormal eye movements as shown in the forms of slow saccade, slow pursuit, fixation instability, and saccadic pursuit (García-Murias et al., 2012; Obayashi et al., 2015). These ataxia signs could be correlated to the dysfunction of the cerebellum as Purkinje cell degeneration was evident in the atrophic cerebella (Ikeda et al., 2012).

### Common Motor Neuron Signs

Patients with SCA36 also demonstrated the signs of motor neuron disease in various ways when the disease progressed. In Ohta’s initial report in 2007 (Ohta et al., 2007), two patients showed tongue atrophy, weakness, fasciculation, and muscular atrophy. Fasciculations and decreased muscle tone were seen in other extremities and the trunk. The muscular atrophy primarily impacted the regions mentioned earlier, with limited impacts toward skeletal muscles in the truncal and proximal regions of the body. However, such muscular atrophy was not seen in other body parts, so the prognosis is different from that in a typical amyotrophic lateral sclerosis (ALS) patient, which usually has broader impacts to all muscles and lead to the death of patients in several years after disease onset (Kobayashi et al., 2011). The affected motor neurons seemed to be limited to the second motor neurons in the lowest part of the brain stem, as shown in Garcia-Murias et al.’s (2012) study. Overactive reflexes (hyperreflexia) could be seen in up to 79% of patients, indicating the impairment of motor neuron function (Ikeda et al., 2012). In a study comparing the swallowing function in typical ALS, SBMA, and SCA36 using the videofluoroscopic swallow (VFS) method, it was found that although SCA36 patients had tongue atrophy, their swallowing frequency and the maximal tongue pressure were comparable to normal controls, while those with ALS and SBMAs were significantly worse (Morimoto et al., 2013), indicating the more preserved muscle function of swallowing in SCA36 patients.

### Other Nervous System Function Impairment

Patients with SCA36 also have other neurologic signs in addition to ataxia and motor neuron signs. These include the following:

Cognitive function decline: A study in twelve SCA36 patients found that these patients had a significant decrease in their frontal executive functions measured by two different ways. Such decline was related to the disease duration and scales of ataxias (Abe et al., 2012). In patients with advanced disease duration, the frontal cortex could also be atrophic.

Hearing impairment: In Garcia-Murias’s study, it was found that some patients had moderate to severe bilateral decline of sensorineural hearing loss, especially above 2,500 Hz (García-Murias et al., 2012). In a 2013 report of evaluating acoustic function in the SCA36 patients, it was found that their pure-tone average (PTA) was significantly decreased compared to normal control and other ataxic groups, especially at high-frequency range of 4,000–8,000 Hz. Some patients showed abnormal brain stem auditory-evoked potentials (BAEPs), indicating the impairment of the inner ear or peripheral part of the auditory system (Ikeda et al., 2013). Of note, the severity of hearing impairment was also correlated with the progression of ataxia.

Dystonia: Miyashiro et al. (2013) reported a case of oromandibular dystonia. This patient showed involuntary dystonic spasms only at the oromandibular region, and this symptom was not responding to sensory tricks. In another case study, Nakazato et al. (2015) reported two siblings with SCA36 showing cervical dystonia.

## Molecular Diagnosis

Once the symptoms of SCA36 are present, the most reliable way of molecular diagnosis of SCA36 is *via* repeat-primed PCR (Kobayashi et al., 2011). Using one fluorescent-labeled primer on one side and the other primer targeting the expanded repeats landing on the hexanucleotide repeat region, PCR amplification would result in amplicons, which would show a typical sawtooth pattern in fragment size analysis. Using this method, the expansion of large expanded repeats can be revealed. The range of sizes of the expanded repeats could be further evaluated *via* the Southern blotting method using probes against the proximity regions of the repeats. Due to the instability of the expansion, such Southern blotting results often show a smeared banding pattern (Kobayashi et al., 2011).

Brain imaging could also be applied in patients with SCA36 to detect structural and functional abnormalities of brains. Magnetic resonance imaging (MRI) of brains from patients with SCA36 can be used in symptomatic patients to validate the atrophy of the cerebella (Kobayashi et al., 2011; Aguiar et al., 2017). The fluorodeoxyglucose-positron emission tomography (FDG-PET) technology could also be applied in GGCCTG repeat expansion carriers from the families of SCA36 patients at asymptomatic and early symptomatic stages (Aguiar et al., 2017). It is interesting that the abnormal cerebellar metabolism can appear early in asymptomatic expansion carriers under 40s, before the onset of ataxia (Aguiar et al., 2017). Such metabolic abnormality could start asymmetrically from the vermis and the right cerebellum, then in both cerebellar hemispheres, and in the brain stem when disease progresses (Aguiar et al., 2017).

## Genetic Anticipation

One important feature of nucleotide repeat expansion diseases is the earlier age of onset (AOO) and severer symptoms in the following generations, which is known as anticipation (Carpenter, 1994). Relationship of the repeat size and the anticipation was evident in diseases with repeat expansion at the coding region like those seen in Huntington’s disease (Trottier et al., 1994). In the case of SCA36, the GGCCTG repeat sizes can be more than 1,000 repeats, and the somatic heterogeneity of the repeat sizes is quite common, so the real relationship of the repeat size and AOO was not well established. One study of a four-generation family specifically reported the earlier age of onset in successive generations of this family (AOO in the first generation at 70s, in the second generation at late 40s, and in the third generation at early 30s), but the data of repeat size were not very convincing due to the technical difficulties with very long repeats (Ohta et al., 2020). Interestingly, three familial patients with short GGCCTG repeats (between 25 and 31 repeats) were identified in three pedigrees in 2015 (Obayashi et al., 2015), and two of them showed later AOO (repeat size 26, AOO 65; repeat size 31, AOO 63), while one showed relatively early AOO (repeat size 25, AOO 44). With the limitation of the available data, more analyses would be needed to make a clear conclusion on the possible genetic anticipation in SCA36.

## Molecular Pathogenesis

Many of the repeat expansion diseases are caused by the translation of the expanded repeats in the coding regions, like those seen in CAG repeats of the polyQ diseases (Orr, 2001). However, for diseases like SCA36, since the expanded repeats are located in the intronic region, which is normally untranslated, there might be some other mechanisms to explain how the repeat expansion contributes to the disease pathogenesis.

**FIGURE 1:**
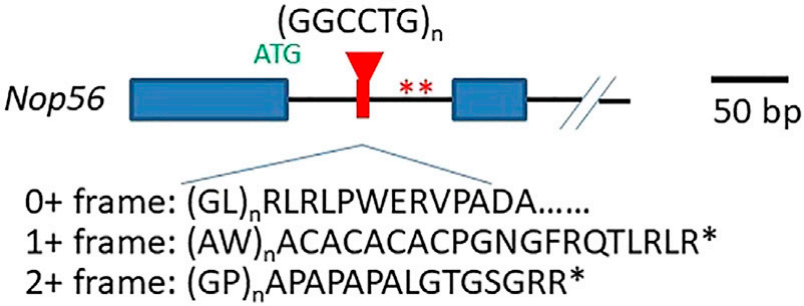
Structure of GGCCTG repeats and its surrounding sequences. The relative location of the repeat expansion in the *Nop56* gene and the unconventional translation products from all reading frames on the sense transcript are shown. Exon 1 and exon 2 are shown as closed blue boxes, and the black solid line indicates the introns. The repeats are in red. The *Nop56* start codon ATG (in green) and the two stop codons in frame of unconventional translation producing AW and GP dipeptide repeats are indicated with two asterisks (in red). The schematic of the *Nop56* gene is drawn on scale with the 50 bp length showed on the right.

**FIGURE 2:**
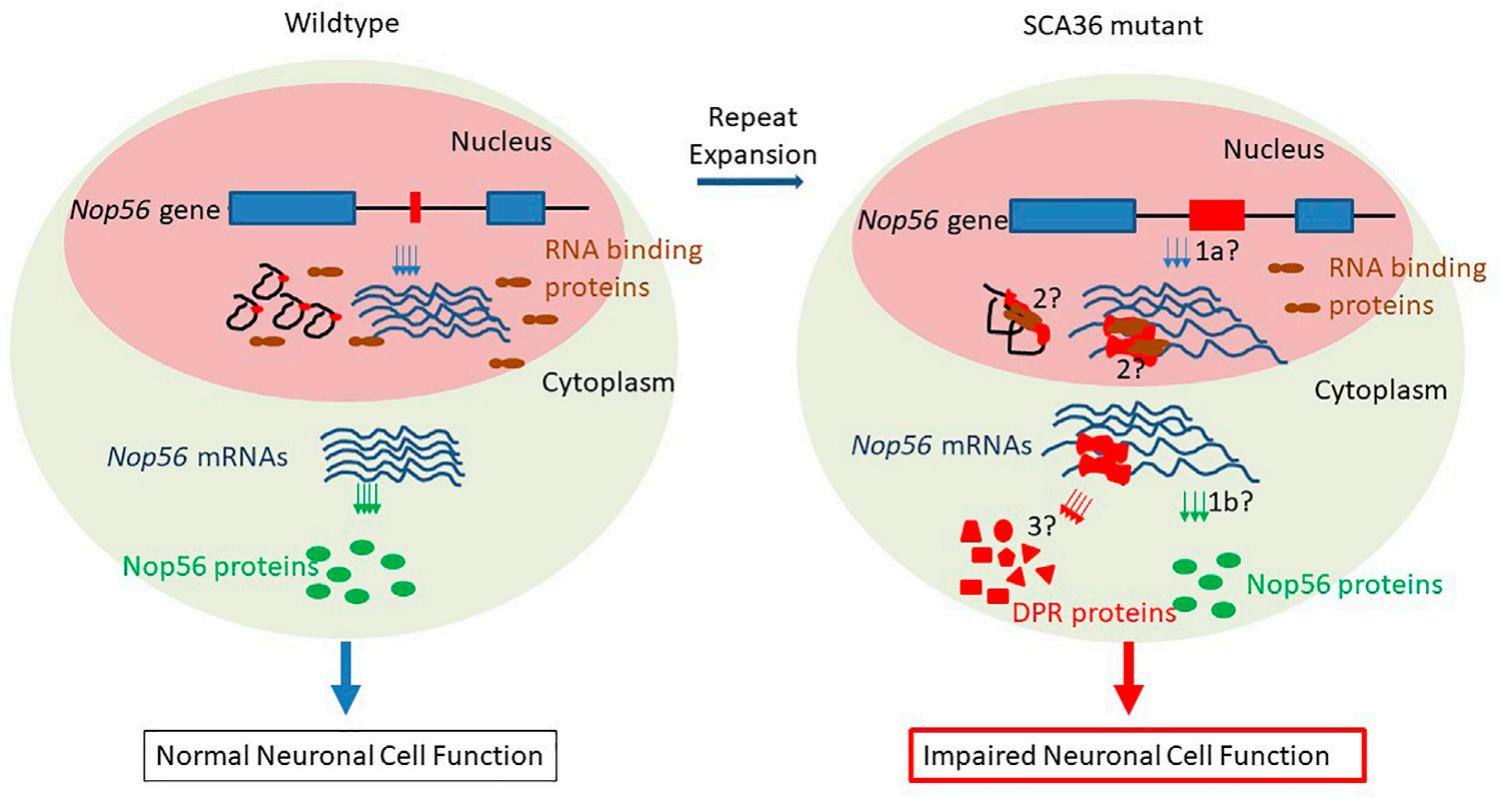
Possible mechanisms of pathogenesis in SCA36. Left panel (normal condition): in the nucleus, the short repeat-containing wild-type *Nop56* genes (exon 1 and exon 2 in blue boxes, black solid line as introns, and GGCCTG repeats in red boxes) are transcribed into mature mRNAs (blue lines), and the repeat-containing introns spliced out as lariats (in black), other RNA-binding proteins (in brown) are scattered in the nucleus; in cytoplasm, *Nop56* mature mRNAs are translated to normal amount Nop56 proteins (green ovals) and neurons are functioning normally. Right panel (SCA36 condition): in nucleus, the mutant *Nop56* gene with expanded GGCCTG repeats (in red box) could be transcribed at different amounts (haploinsufficiency mechanism/1a), and the expanded GGCCUG repeat RNA (in red) could be either present as lariats or retained in mRNAs to sequester other RNA-binding proteins (RNA gain-of-function mechanism/2); in the cytoplasm, the expanded GGCCUG repeat-containing *Nop56* mRNAs could either impede the production of NOP56 proteins (haploinsufficiency mechanism/1b) or undergo unconventional translation (RAN translation mechanism/3) into dipeptide repeat (DPR) proteins (red triangle, rectangle, circle, pentagon, and trapezoid). These possible mechanisms result in the impaired neuron functions ultimately.

*Nop56* gene haploinsufficiency hypothesis: the expanded GGCCTG repeats are located in the first intron of the host gene *Nop56* (Figure 1), so it could potentially impact the transcription of *Nop56* mRNAs (Figure 2, process 1a) or the translation of Nop56 protein (Figure 2, process 1b) in the mutant allele copy with repeat expansion. The host gene *Nop56* encodes a 594 amino acid-long ubiquitously expressed protein, which has conserved function as one of the core scaffold proteins of the box C/D small nucleolar ribonuclear protein (snoRNP) complex seen from yeast to human beings (Gautier et al., 1997). Particularly, the Nop56 protein locks and stabilizes the box C/D snoRNP complex (Bizarro et al., 2014), which is known to catalyze the 2′-O-methylation of ribosomal RNAs (reviewed in Watkins and Bohnsack, 2012). To answer whether the expansion altered the *Nop56* gene expression level or not, Kobayashi *et al.* examined the changes of *Nop56* mRNAs or proteins in patient’s lymphoblastoid cell lines (LCLs), but no significant changes were found in either mRNAs or proteins (Kobayashi et al., 2011). The nuclear structure Cajal body, where box C/D snoRNPs reside for rRNA modification, did not seem to be reduced either (Kobayashi et al., 2011). These findings were not able to support the haploinsufficiency hypothesis.

Gain-of-function hypothesis of repeat-containing RNAs: the transcripts from expanded repeats located at 3′- or 5′-untranslated regions often accumulate to form aggregate-like structure or RNA foci. Such aggregate can sequester some RNA-binding proteins, as seen in the disease of myotonic dystrophy type 1 (Lee & Cooper, 2009). In SCA36 patient neurons, the first intron containing expanded GGCCUG repeats in pre-mRNAs could either be spliced out and remained in the intron lariats or retained, resulting in abnormal mRNAs containing the GGCCUG repeats, both scenarios could lead to the formations of RNA foci and the sequestrations of GGCCUG-specific RNA-binding proteins (Figure 2, process 2). It seemed to be clear that these expanded repeat-containing RNAs did form such foci and could sequester RNA-binding proteins like SRSF2 and other proteins (Kobayashi et al., 2011). These RNA foci were common in neuronal nuclei of the cerebrum, cerebellum, inferior olive, spinal cord, and temporal muscle. The size of such RNA foci could be variable, and some giant foci can be seen in Purkinje cells, spinal motor neurons, and most frequently in the inferior olivary nucleus, suggesting the connection between the RNA foci formation and the dysfunction of neurons (Liu et al., 2014). However, understanding exactly whether or how such RNA foci participate in the pathogenesis still requires more data from future research.

Unconventional translation hypothesis: repeat-associated non-ATG-driven (RAN) translation through the expanded repeats was initially found in SCA8 (reviewed by Zu et al., 2011), and it was accepted to be one of the major mechanisms in the pathogenesis of GGGGCC repeat expansion-related C9ALS/FTD (Mori et al., 2013) (also reviewed in Freibaum and Taylor, 2017). In C9ALS/FTD, the expanded GGGGCC repeats can be possibly transcribed from both directions, producing sense and antisense transcripts, and the RAN translation from all three reading frames would result in five different dipeptide repeats (DPRs), and these DPRs can elicit cytotoxicity *via* various ways (Freibaum and Taylor, 2017). Since the nature of the hexanucleotide repeat is very close in these two diseases (GGCCTG vs. GGCCGG), the mutant allele might also produce both sense and antisense transcripts with the potential to undergo RAN translation through the expanded repeat region (Figure 2, process 3). From the expanded repeat region of sense transcript, three different DPRs could be produced: glycine–proline (GP), possibly with the additional help of an AUG codon, while glycine–leucine (GL) and alanine–tryptophan (AW) *via* such unconventional translation (Figure 1). Two groups investigated the possible translations using the expanded repeats and compared the divergence of the pathogenesis in both C9ALS/FTD and SCA36 mouse models expressing these repeats (McEachin et al., 2020; Todd et al., 2020). Both reports confirmed the presence of expanded repeat-translated DPR products, like the glycine–proline (GP) in the GGCCTG repeat-expressing mice neurons. Interestingly, the pattern of the affected brain area was more concentrated to the cerebellum in the GGCCTG repeat-expressing mice, distinct from the cortical neurons seen in GGGGCC repeat-expressing mice (Todd et al., 2020). The GP dipeptide level was higher in the SCA36 mice model. More importantly, the status of GP DPR proteins stayed mostly in soluble status in the SCA36 model, whereas such GP DPR proteins were largely seen as aggregates in the C9ALS/FTD model. It is still not very clear whether this phenotypic divergences in these mice models of C9ALS/FTD and SCA36 were due to the differences in the nature of the RNA structure or the nature of DPR species from all reading frames of both sense and antisense transcripts (GP, GA, PA, GR, and PR in C9ALS/FTD vs. GP, AW, GL, AQ, and PR in SCA36), which may require further in-depth investigation.

## Attempts of Therapeutic Interventions

SCA36 is a slowly progressive disease with multiple symptoms. Since there is no cure available at the moment, supportive treatments for some symptoms were recommended (https://www.ncbi.nlm.nih.gov/books/NBK231880/). Several pilot studies have been performed to explore the potential treatment for this disease.

In 2013, Miyashiro et al. (2013) used botulinum A toxin injection to manage the effects of oromandibular dystonia associated with SCA36, but the impact of other symptoms was not mentioned.

Small molecules targeting the expanded repeats could be one strategy to treat this disease. *In silico* studies indicated both GGCCTG repeats seen in SCA36, and GGGGCC repeats seen in C9ALS/FTD can form a regional DNA/RNA structure called guanine quadruplex (Zhang et al., 2018). These structures in GGGGCC repeats can be interrupted by cationic porphyrin TMPyP4 (Zamiri et al., 2014; Alniss et al., 2018). Hirayanagi et al. (2021) tested the porphyrin derivatives and identified two derivatives, namely, sodium copper chlorophyllin and hemin chloride, that could suppress the RNA foci formation in the cell model of SCA36 and that reduced RNA-mediated cytotoxicity.

Antisense oligo (ASO) nucleotides targeting the diseased gene transcripts carrying expanded repeats could be another potential strategy (Silva et al., 2020)). In the C9ALS/FTD scenario, ASOs targeting GGGGCC sense or antisense were carried out under various conditions. ASOs targeting the expanded GGGGCC transcripts in patient fibroblasts and induced pluripotent stem cells (iPSCs) or iPSC-derived neurons (iPSNs) could greatly reduce the RNA foci and reduce the cytotoxicity in the iPSNs (Donnelly et al., 2013; Lagier-Tourenne et al., 2013; Sareen et al., 2013). In a mouse model of C9ALS/FTD expressing the expanded GGGGCC repeats, the ASO targeting the expanded GGGGCC repeat transcripts could effectively reduce the RNA foci and the DPR production in a sustained manner even with one dose treatment (Jiang et al., 2016). Based on these findings, a phase I clinical trial using ASO BIIB078 against C9ALS/FTD was actively carried out (https://clinicaltrials.gov/ct2/show/NCT03626012). In the case of SCA36, Matsuzono et al. (2017) found that 2′-O, 4′-C-ethylene-bridged nucleic acid ASOs against expanded GGCCUG repeats-containing transcripts at different sites can all reduce the RNA foci in SCA36 patient iPSC and iPSNs Some of these ASOs targeting directly against the expanded GGCCUG region had no impact of *Nop56* mRNA in these cells. In another study, McEachin et al. (2020) used ASOs targeting the first introns at the expanded GGCCUG repeat region or the upstream of the expanded repeats. It turned out that at optimal concentrations, both ASOs could effectively reduce the poly(GP) DPR level, without negatively impacting the Nop56 protein level in the fibroblasts and lymphoblastoid cell lines (LCLs) from SCA36 patients.

## Conclusion and Future Directions

SCA36 is a new member of SCAs that occurs preferably in people from select regions of Japan and Spain and their descendants, while patients show signs of typical ataxia, and the symptoms due to the dysfunction of motor neurons are apparent when the disease progresses. The expanded repeats in the first intron of the host gene *Nop56* can undergo unconventional translation, and the resulting DPR products could be the important player of disease pathogenesis. As there are currently no effective treatments for this disease, the development of ASOs with promising results in iPSCs gave the patients some hope to possibly prevent the progression of this disease. It should be noted that given the complex nature of human diseases, the development of ASOs in treating nucleotide repeat expansion diseases including SCA36 would still have a long way to go, as exemplified by the recent failure of the phase 3 clinical trial of ASO product tominersen against HD (Kwon, 2021). So, we need to remain patient while being optimistic. It would be also very interesting to see whether certain small molecules like derivatives from TMPyP4 can be developed to target the clearance or the degradation of the toxic expansion-containing RNAs or DPRs as the administration of such drugs can be easier to maneuver than the usage of ASOs.
